# Pyrrolidinium Containing Ionic Liquid Electrolytes for Li-Based Batteries

**DOI:** 10.3390/molecules25246002

**Published:** 2020-12-18

**Authors:** Louise M. McGrath, James F. Rohan

**Affiliations:** Electrochemical Materials and Energy Group, Tyndall National Institute, University College Cork, Lee Maltings, T12 R5CP Cork, Ireland; louise.mcgrath@tyndall.ie

**Keywords:** anode, cathode, electrolyte, energy storage, ionic liquids, lithium ion batteries, lithium metal batteries, polymer gel electrolyte, pyrrolidinium

## Abstract

Ionic liquids are potential alternative electrolytes to the more conventional solid-state options under investigation for future energy storage solutions. This review addresses the utilization of IL electrolytes in energy storage devices, particularly pyrrolidinium-based ILs. These ILs offer favorable properties, such as high ionic conductivity and the potential for high power drain, low volatility and wide electrochemical stability windows (ESW). The cation/anion combination utilized significantly influences their physical and electrochemical properties, therefore a thorough discussion of different combinations is outlined. Compatibility with a wide array of cathode and anode materials such as LFP, V_2_O_5_, Ge and Sn is exhibited, whereby thin-films and nanostructured materials are investigated for micro energy applications. Polymer gel electrolytes suitable for layer-by-layer fabrication are discussed for the various pyrrolidinium cations, and their compatibility with electrode materials assessed. Recent advancements regarding the modification of typical cations such a 1-butyl-1-methylpyrrolidinium, to produce ether-functionalized or symmetrical cations is discussed.

## 1. Introduction

Due to advancements in the fields of electronics, and the deployment of wireless communication systems, mobile devices and ubiquitous services, the Internet of Things (IoT) has been able to step out of its infancy, and is constantly gaining traction, with current trends suggesting that 75.44 billion IoT devices should be connected by 2025 [1]. This progress has been realized due to the integration of several enabling technologies (energy harvesters, sensors and energy storage), which can create autonomous devices, which can then be deployed and forgotten.

However, one of the main constraints to the deployment of IoT devices is the power requirement. Energy harvesters alone are rarely sufficient to power the IoT devices, due to their limitations, e.g., availability of light for photovoltaic cells. Therefore, the coupling of energy storage devices with appropriate power management systems is required. Advancements in sensor technologies are leading to rapid miniaturization, which is hindered by the large energy storage devices. This highlights the need for rapid development of miniaturized energy storage devices with a high power capability for sense, actuation and communication [2].

A crucial hurdle towards the development of feasible microbatteries is that the charge capacity decreases with size. The decrease in energy originates from the fact that the capacity of a battery scales linearly with the volume of the electrodes. Therefore, the volumetric capacity of the electrode material is more important than the gravimetric capacity [3,4,5]. The most commonly investigated microbattery technology relies on lithium cobalt oxide (LCO) materials coupled with Li metal and a solid-state electrolyte. The scarcity of cobalt (Co) [6] and its price instability [7] make it unattractive for continuous use, and researchers are investigating electrode materials with reduced Co content or are entirely Co-free. High operational temperatures are required for most microbatteries without LCO cathodes due to the low ionic conductivity of the compatible electrolyte material and sluggish Li^+^ diffusion [8,9,10,11,12,13,14,15,16,17,18].

There is a need to identify potential alternative electrolytes with high power capability beyond what is possible with present day inorganic solid-state materials. The objective of this review is to provide a comprehensive analysis of ionic liquid electrolytes, particularly pyrrolidinium-based ILs. Firstly, a brief discussion of various IL cations, which can be utilized for energy storage applications is presented. An in-depth analysis of pyrrolidinium-based ILs is given due to their superior versatility when compared to other ILs such as imidazolium. The ethyl-, propyl- and butyl- analogues of pyrrolidinium ILs have shown applicability for energy storage applications each with different physico- and electrochemical properties. Their compatibility with a variety of anion materials is discussed. The formation of polymer gel analogues is outlined where applicable, and the liquid and polymer gel half- and full-cell compatibility with a wide variety of electrode materials is shown. Functionalized pyrrolidinium-based electrolytes are also presented and contrasted with their non-functionalized counterparts.

## 2. Ionic Liquids (IL) for Energy Storage Applications

Examples of ILs that have been identified as suitable electrolytes include: ammonium [19,20,21,22,23,24,25,26,27,28,29,30,31,32,33,34,35,36,37,38,39,40,41,42,43,44,45,46], pyridinium [47,48,49,50], piperidinium [51,52,53,54,55,56,57,58,59,60,61,62,63,64,65,66,67,68,69,70,71,72,73,74,75,76,77], imidazolium [78,79,80,81,82,83,84,85,86,87,88,89,90,91,92,93,94,95,96,97] and pyrrolidinium. Each cation offers favorable properties, for example, piperidinium ionic liquids possesses non-flammability, large electrochemical window (−3.8 to 2.5 V vs. Pt) [98], and inherent conductivity, which make them suitable for use as an electrolyte solvent in electrical energy storage devices [51,52].

Numerous studies have been carried out to determine the most suitable cation-type for use with Li-based batteries. Ammonium cations, particularly the liquid analogue, are suitable for Li and Li-ion batteries as they show good cyclability with various cathode and anode materials in both thin-film and nanostructured configurations. In some instances, full cell capabilities have been demonstrated incorporating high capacity anode materials such as Si, thus showing their suitability as liquid electrolytes [29]. Unfortunately, there is little literature available for the polymer gel analogues, with the exception of TMHA-TFSI [31] and DEME-TFSI ILs [40,41,42,43], which limits its use with solid-state batteries. In addition, high operation temperatures (>60 °C) are required in order for appreciable capacities to be obtained using the polymer gel analogue.

Pyridinium-based [47,48,49,50] ILs have been utilized as a solid state electrolyte (polymer gel form) where Gupta et al. [47] and Yang et al. [49] synthesized promising polymer gel electrolytes, which offer good cyclability with lithium iron phosphate (LFP) electrodes and high coulombic efficiencies. To the best of our knowledge, no other research has been carried out with other cathode or anode materials, which may suggest that the compatibility with other electrode materials may be limited, therefore hindering its use as an electrolyte for Li-ion batteries.

The focus of piperidinium-based ILs is mainly concerned with *N*-propyl-*N*-methylpiperidinium (PP_13_)-based electrolytes, due to its compatibility with various electrode materials at different temperatures [51,52,53,54,55,56,57,58,59,60,61,62,63,64,65,66,67,68,69,70,71,72,73,74,75,76,77]. However, further research is required to advance the utilization of PP_13_TFSI in both its liquid and polymer gel analogue forms, in addition to a multitude of other piperidinium-based ILs [99,100,101,102,103,104,105,106,107]. Other piperidinium cations are not as extensively studied, however, they were investigated with a range of cathode and anode materials across a wide variety of temperatures (room temperature to 150 °C).

Extensive research has been carried out on 1-ethyl-3-methylimidazolium-based ILs. This IL has shown compatibility with a wide range of cathode and anode materials in both its liquid [78,79,80,81,82,83,84,85,86,87] and polymer gel form [88,89,90,91,92,93,94,95,96,97]. Other imidazolium-based ILs have been synthesized and characterized, and their physicochemical properties were determined [108,109,110]. More recently, functionalized-imidazolium-based ILs are being investigated including phosphonate- [111], and Si-functionalized ILs [112,113]. 

## 3. Overview of Ionic Liquids

Ionic liquids offer a unique set of properties that make them important candidates for a number of energy related applications. ILs are salts, whose ions are poorly coordinated, thus resulting in their solvent-like appearance at temperatures below 100 °C [114]. They have garnered interest as alternatives to both organic and solid-state electrolytes because of their tunable properties, which depends upon the cation and anion combination. They can be tuned to exhibit low volatility coupled with high ionic conductivity and electrochemical and thermal stability. It is therefore possible to design electrolytes, for specific applications such as, 1-butyl-1-methylpyrrolidinium bis(trifluoromethylsulfonyl)imide, which exhibits high lithium ionic conductivity (3 mS cm^−1^) at room temperature for energy storage [115].

Diffusion within ILs is unique as fluid mechanic equations under simplified conditions are not applicable [116,117,118,119,120,121,122,123,124], for example, diffusion in ILs is similar to that in solid-state materials [116,117,118] but further complicated by the ever-changing diffusion pathways caused by the ion pairings [119,120,121,122,123,124]. ILs are often viscous fluids and a direct channel for diffusion is not provided, in conjunction with blocking interactions between the electroactive species and IL ions. For these reasons, conventional one-dimensional diffusion as observed in organic electrolytes cannot be assumed. Diffusing ions can collide with IL ions and either be deviated from the straight pathway or deflected. Therefore, it has been suggested that the viscosity of the ILs is not the primary factor for slow diffusion but the active interaction of Li^+^ ions in the structure of the ILs [125].

As with organic solvents the electrochemical reduction of the IL cation can result in the formation of a solid-electrolyte interphase (SEI) layer, however, the anion can also contribute to its formation. The anion plays a substantial role in the electrochemical stability of the IL, which in turn affects that electrochemical stability window (ESW). Sun et al. [126] found that the ESW of 1-ethyl-3-methylimidazolium ([emim]^+^) based ILs with different aprotic heterocyclic anions is highly dependent on the anion. Hayyan et al. [127] investigated different cation/anion combinations to determine the effect of the IL structure on its ESW. They determined that certain cations such as phosphonium (P_14,666_^+^) have better reductive stability than pyrrolidinium and imidazolium. Similarly, certain anions (TFSI^−^) offer significantly better oxidative stability than others (e.g. OTf^−^). Understanding the reductive and oxidative limits of cations and anions permits the correct combination to be chosen, in addition to tailoring the combination to suit specific potential needs. 

Another parameter to consider is the availability of a “free” proton on the cation of certain ILs. ILs whose cations have this “free” proton are called protic ILs, while those without are referred to as aprotic ILs (AIL) [128]. Both offer the favorable characteristics associated with ILs such as negligible vapor pressure, non-flammability and high chemical and thermal stabilities [129,130,131] and some also exhibit high ionic conductivity [129,130,131] but AILs are utilized more frequently as electrolytes for lithium-based batteries due to their favorable properties such as wider ESW when compared to their protic counterparts [132]. 

This review will focus on one particular family of AIL cations, the pyrrolidinium cations, due to their versatility and compatibility with Li-based battery materials. They enable the use of different cations including ethyl-, propyl- and butyl-, in addition to ether functionalization. The structure and name of the cations that will be discussed in this review are outlined in Table 1.

## 4. *N*-ethyl-*N*-methylpyrrolidinium (C_2_mpyr)-Based ILs

*N*-ethyl-*N*-methylpyrrolidinium (C_2_mpyr)-based ILs are solids at room temperature [133], much like other ILs consisting of ethyl-based cations [53,134]. C_2_mpyr-based ILs have hindered rotation about the axis, which leads to increased viscosity (9.3 cP vs. 8.3 cP and 8.6 cP at 90 °C) when compared to propyl or butyl side chains, respectively [133]. Many of the electrolytes containing C_2_mpyr-cations are polymer gel based rather than liquid, where to date, the most commonly used anion for C_2_mpyr-based electrolytes is bis(fluorosulfonyl)imide (FSI^−^), with either LiTFSI or LiFSI salt. 

### 4.1. PVDF-HFP/PEO Containing Polymer Electrolytes

Conventional polymer matrices such as PVDF have been investigated with C_2_mpyrFSI and LiFSI salt [135,136]. The optimum LiFSI concentration was determined to be 10 wt % as it offered a high ionic conductivity (10^−6^ S cm^−1^ vs. 10^−7^ S cm^−1^), when compared to 1 wt % LiFSI. The effect of PVDF concentration was also investigated as it interacts on a molecular level with C_2_mpyrFSI, which leads to increased molecular disorder [135]. The optimum PVDF content was determined to be 10 wt % as it provided the best thermal and ionic conductivity properties. Compatibility with Li metal was shown using a combination of cycling and EIS analysis. At higher LiFSI concentrations (>10 wt %), a continuous increase in the interfacial resistance was observed, which is indicative of continuous SEI layer formation. For all investigated salt concentrations, cracks eventually formed on the surface of Li metal, which allowed for the formation of dendrites, however, this occurred after hundreds of cycles. Further optimization of the Li/electrolyte interface may mitigate the dendrite formation, which could allow for the creation of a C_2_mpyrFSI-based microbattery as the polymer gel exhibited compatibility with LiNi_0.3_Mn_0.3_Co_0.3_O_2_ (NMC) [136]. A high cut-off voltage of 4.6 V was utilized where an initial discharge capacity of 120 mAh g^−1^ was obtained, which decreased over 30 cycles due to chemical and structural changes [137], despite this, coulombic efficiencies were maintained above 90% over the course of cycling. Its performance with NCM is comparable to a conventional electrolyte (1 M LiPF_6_ in EC/DEC (1:1)) [136].

### 4.2. Other Polymer Matrices 

**Figure 1:**
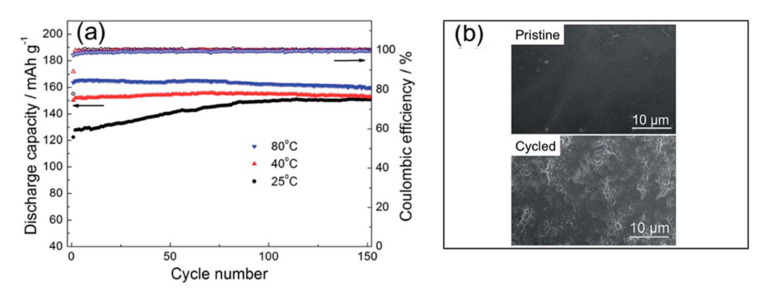
The performance of Li|SPE: 50:20:50 P(DADMA)-TFSI:LiTFSI: C_2_mpyrFSI|LiFePO_4_ cells: (**a**) discharge capacity and coulombic efficiency versus cycle number at 25 °C, 40 °C and 80 °C at a current rate of 0.2 C, (**b**) SEM images of the pristine lithium and lithium anodes after a rate test (0.2 C, 0.5 C, 1 C and 0.2 C). Adapted from Li et al. [140] with permission from The Royal Society of Chemistry.

Other authors have shown the viability of C_2_mpyr-based IL electrolytes when utilizing a polymeric ionic liquid (PIL) rather than conventional polymer matrices, due to their higher ionic conductivities in addition to better film forming properties and desirable electrochemical properties [138,139]. Li et al. [140] utilized the PIL: poly(diallyldimethylammonium) bis(trifluoromethylsulfonyl)imide (P(DADMA)-TFSI) with C_2_mpyrFSI and LiTFSI salt. An ionic conductivity of 1.54 × 10^−4^ S cm^−1^ at 25 °C was obtained for a 50:20:50 P(DADMA)-TFSI:LiTFSI: C_2_mpyrFSI ratio. This value is significantly improved when compared to the electrospun PVDF-based polymer gel electrolytes by Zhou et al. [135,136]. Compatibility with Li metal was exhibited with 1000 cycles achieved in a symmetrical cell without short circuiting. In addition, good electrochemical performances were noted with LFP across a variety of temperatures (25 °C, 40 °C and 80 °C) with initial discharge capacities and coulombic efficiencies of 122.3 mAh g^−1^ with 77.8%, 150.2 mAh g^−1^ with 89.2% and 163.6 mAh g^−1^ with 97.6%, respectively, being obtained at 0.2 C. All cells maintained their capacity over the course of 150 cycles, with an improvement in the capacity for the cell cycled at 25 °C. Unlike Zhou et al. [135,136], dendrite formation was hindered within the P-DADMA-TFSI-based electrolyte, however, post-mortem analysis revealed the presence of pits on the surface of the Li metal electrode, which may yield dendrites upon prolonged cycling as shown in Figure 1 [135,140].

### 4.3. Encapsulation of Electrode Particles

**Figure 2:**
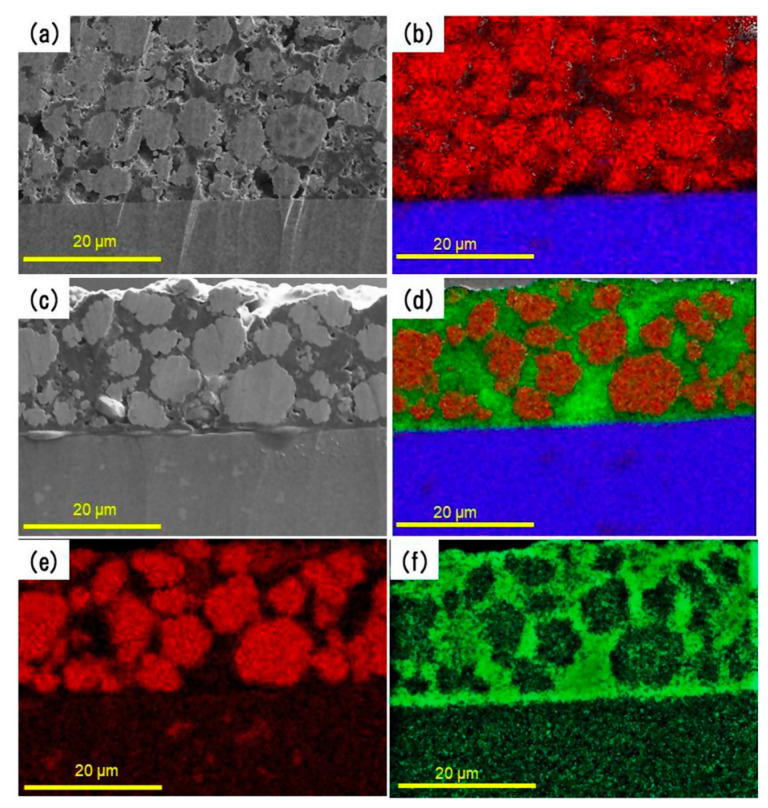
SEM and EDX-mapping images of the sectional view of the NMC cathode. (**a**,**b**) are with no ionogels (control sample) and (**c**,**d**) are with ionogel respectively. Red, green and blue colors indicate cobalt (NMC), sulphur (ionogels) and aluminum (current collector), respectively. (**e**,**f**) are EDX-mapping images of cobalt and sulphur extracted from (**d**). Reprinted with permission from Ogawa et al. [32].

The utilization of C_2_mpyr-based ILs is not limited solely to polymer gel electrolytes that are sandwiched between two electrodes as other research groups have investigated the in-situ formation of the ionogel electrolyte within an electrode to ensure encapsulation of the electrode particles [32]. SEM and EDX analysis of the electrodes was used to show the penetration of the ionogel into the electrode as the electrode particles were encapsulated by the ionogel (Figure 2). This is a viable method for ensuring high surface contact between the gel-based electrolyte and the electrode, particularly where particle or nanostructured materials were utilized. A full cell was assembled by Ogawa et al. [32] to show the enhanced capabilities of the encapsulated cathode and anode particles. The full cell consisted of an LTO anode and NMC cathode. The internal resistance of the battery plays a significant role in full cell performances. Ogawa et al. [32] showed a significant increase (~0.34 mAh) in the batteries discharge capacity when the C-rate was decreased from 0.05 to 0.01 C. These results pave the way for further analysis of polymeric ionic liquid-based electrolytes as they can be synthesized using solvent free processes and have high wettability of the electrode material.

### 4.4. Alternative Cation/Anion Combinations

In addition to the utilization of PILs, further research is being carried out to determine alternative cation–anion combinations of the C_2_mpyr-based electrolytes, which may yield more favorable properties than typical FSI- and TFSI-based ILs. Recently, Yamaguchi et al. [141] investigated the effect of various anions on the properties of the IL salt, as it has been reported that FSI-based salts offer higher ionic conductivities (1.23 × 10^−6^ S cm^−1^) than TFSI-based salts [142]. In addition, high ionic conductivities of 1 × 10^−3^ S cm^−1^ have been reported for carbamoylcyano(nitroso)methanide (ccnm) anions in combination with C_2_mpyr cations [143]. Yamaguchi et al. [141] determined that the ionic radius ratio (*ρ* = r_small ion_/r_large ion_ < 1) can have an effect on the ionic conductivities. Lower *ρ* values exhibited higher ionic conductivities, which is attributed to the size and concentration of vacancies within the crystal [144,145,146], therefore, lower *ρ* values have more voids due to large differences in the ionic radii. This allows for greater tunability of the IL, whereby new battery electrolytes can be considered with anions different to typical FSI or TFSI-based anions. Further work is required before new anions can be realized for battery applications, however, these results show that favorable thermal and ionic conductivity properties can be attained using unconventional anions.

To date, limited cathode and anode analysis has been carried out with only NCM, LFP, LTO and Li metal having exhibited compatibility with the C_2_mpyr-based electrolytes. The primary cation/anion combination for C_2_mpyr-based electrolytes is C_2_mpyrFSI with little literature dedicated to C_2_mpyrTFSI-based electrolytes. Further investigations into different C_2_mpyr/anion combinations may lead to next generation battery electrolytes, however, in order to realize microbatteries, other pyrrolidinium-based ILs whose physiochemical properties have been thoroughly characterized need to be investigated.

## 5. *N*-propyl-*N*-Methylpyrrolidinium (C_3_mpyr)-Based ILs

Characterization of *N*-propyl-*N*-methylpyrrolidinium (C_3_mpyr)-based ILs have been carried out by many research groups to determine the physiochemical properties of the IL with various anions [147,148]. The biodegradability and solubility of C_3_mpyr-based ILs, and other pyrrolidinium-based ILs have also been investigated [149]. Thermal stability analysis has been carried out using commercial electrode materials (NCM, LFP, graphite, etc.) with LiFSI salt in C_3_mpyrFSI electrolytes [150]. In addition to thermal stability, the total heat generated by the electrode was determined at various states of charge [150]. This study also showed that certain cathode and anode materials might undergo exothermic reactions at different temperatures, therefore, indicating the need to select the correct electrode material combinations for high temperature applications. The characterization of C_3_mpyr-based electrolytes also extends to its compatibility with different types of separators [151,152], particularly when Li metal anodes are utilized. The long-term cyclability of Li metal based cells is affected by the separator and small pore sizes (0.06 µm) can prevent short circuits by inhibiting Li dendrite penetration of the separator [152]. Further information regarding different separator materials can be found in the review by Francis et al. [151].

### 5.1. Liquid Electrolyte

#### 5.1.1. C_3_mpyrFSI/Cathode Compatibility

Compatibility with various cathode and anode materials were shown in C_3_mpyr-based electrolytes. In some cases, the C_3_mpyr-based electrolytes were utilized as an interlayer [153], however much of the research has focused on its use as an electrolyte. Two cation/anion combinations have been extensively investigated, namely, C_3_mpyrFSI and C_3_mpyrTFSI. For the conventional cathode materials such as NMC and LFP electrodes, analysis studies have been carried out primarily with C_3_mpyrFSI and either LiTFSI or LiFSI salt. NMC electrodes have exhibited electrochemical performances comparable to organic electrolytes such as 1.0 M LiPF_6_ in EC:DEC, (1:1) particularly at low C-rates [154].

**Figure 3:**
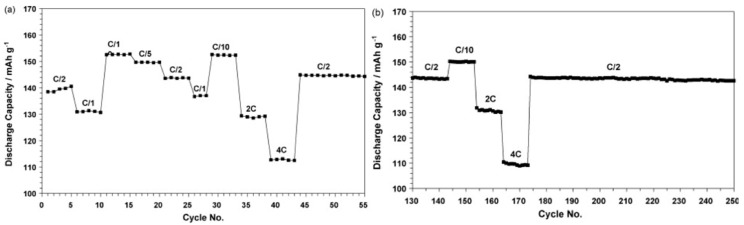
Cycling performance of Li|C_3_mpyrFSI–LiTFSI (0.5 mol kg^−1^) |LiFePO_4_ cell at indicated discharge rates (at 50 °C) for: (**a**) cycles 1–55 and (**b**) cycles 130–250. Charging rate is constant at C/10. Reprinted with permission from Lewandowski et al. [155].

Much like the NMC electrodes, LFP electrodes have also been investigated with C_3_mpyrFSI-based electrolytes. For example, Lewandowski et al. [155] reported that the combination of C_3_mpyrFSI and 0.5 mol kg^−1^ LiTFSI has been shown to work effectively as an electrolyte solution with a metallic lithium anode and LFP cathode. Even at a 4 C rate, a discharge capacity of 110 mAh g^−1^ was obtained at 50 °C, which shows the excellent cyclability of the C_3_mpyrFSI electrolyte with the LFP cathode (Figure 3).

#### 5.1.2. C_3_mpyrTFSI and C_3_mpyrFSI Electrolyte Mixtures

Electrolyte mixtures consisting of C_3_mpyrTFSI and C_3_mpyrFSI have been investigated with NMC and LFP electrodes, whereby different conducting salts were utilized. In the case of NMC, the salt was LiTFSI and the optimum ratio of C_3_mpyrTFSI:C_3_mpyrFSI was determined to be 3:6 [156]. When LiFSI salt is utilized the optimum ratio of C_3_mpyrTFSI:C_3_mpyrFSI was determined to be 1:1 with LFP electrodes [157]. Due to the different properties of the conducting salts, the optimum ratios were chosen based on a compromise between fast ion transport properties, electrochemical/thermal stability, good film forming ability and cost [156]. Huang et al. [157] showed that the ternary electrolyte exhibited the best long-term cyclability when compared to its binary analogues: 0.5 M LiFSI in C_3_mpyrFSI, 0.5 M LiTFSI in C_3_mpyrTFSI and 0.5 M LiFSI in C_3_mpyrTFSI at room temperature, and sub-zero temperatures. This paves the way for low-temperature application electrolytes. Further work to optimize the ratio of C_3_mpyrTFSI to C_3_mpyrFSI, could lead to improvements in the electrode capacity obtained at both elevated and low temperatures, for binary mixtures such as those tested by Lewandowski et al. [155] with higher electrode capacities at C-rates such as 4 C.

#### 5.1.3. C_3_mpyrTFSI/Cathode Compatibility

The studies described above are for FSI-based electrolytes, however analysis of other cathode materials is focused primarily on C_3_mpyrTFSI-based electrolytes. The results obtained in this electrolyte are promising as the electrodes exhibit high electrode capacities and coulombic efficiencies. One such example is a composite cathode material: 0.3Li_2_MnO_3_-0.7LiMn_1.5_Ni_0.5_O_4_ [158], whereby the IL-based electrolyte exhibited a superior performance when compared to organic based electrolytes, 332 mAh g^−1^ and 310 mAh g^−1^_,_ respectively. Even at high current densities such as 750 mA g^−1^ the IL outperformed the organic electrolyte due to the mitigation of Mn dissolution. Nickel sulphide, has also attracted a lot of attention due to its high theoretical capacity (590 mAh g^−1^ for NiS, and 462 mAh g^−1^ for Ni_3_S_2_) [159]. The NiS-Ni_7_S_6_ material exhibited the best CV performance when utilized with 1 M LiTFSI in C_3_mpyrTFSI as stable cycling was observed in addition to higher electrode capacities (450 mAh g^−1^) when compared to an organic electrolyte (200 mAh g^−1^). Studies with vanadium pentoxide (V_2_O_5_) have also indicated promising electrochemical performances as different morphologies including nanoribbon, nanowire, microflake and commercial powder were investigated with C_3_mpyrTFSI [160]. Each morphology yielded greater specific capacity when compared to a conventional organic electrolyte (1 M LiPF_6_ in EC/DMC (1:1)).

#### 5.1.4. Anode Compatibility

**Figure 4:**
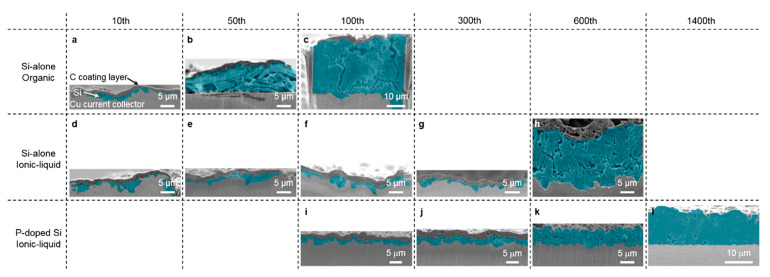
Cross-sectional SEM images of lithiated Si active material layers with a charge capacity limit of 1000 mAh g^−1^. The blue colored area indicates Si, while the topmost layer is a carbon coating. Reprinted with permission from Domi et al. [164]. Copyright 2019 American Chemical Society.

Conventional anode materials such as graphite [156,161,162] are compatible with the C_3_mpyr-based ILs. Alternative higher energy density anode materials such as Si have also been investigated. Recent research is focused on coated or doped Si films to improve the cyclability of the Si electrodes by buffering or mitigating the expansion and contraction effects upon cycling with Li. Superior reversible cycling of graphene coated Si nanoparticles in C_3_mpyrFSI-based electrolyte when compared to the organic electrolytes was shown by Park et al. [163]. The electrodes cycled with the IL electrolyte maintained a capacity of 1800 mAh g^−1^ after 200 cycles, while capacities less than 500 mAh g^−1^ were obtained in the organic electrolyte. A comparison of doped and undoped-Si in 1 M LiFSI and C_3_mpyrFSI electrolytes determined that P-doping led to reduced vertical electrode expansion during cycling with the IL electrolyte as shown in Figure 4 [164], with superior cyclability (>1400 cycles) when compared to its counterpart within an organic electrolyte. The reduction in vertical expansion is attributed to the film’s preference for horizontal expansion due to the film being perpendicularly pressed to the Cu current collector by the separator in the coin cell.

The choice of IL anion can affect the electrochemical performance of P-doped Si electrodes. In C_3_mpyrFSI-based electrolytes stable cycling for over 1400 cycles, with a discharge capacity of 1000 mAh g^−1^ was reported, while the TFSI-counterpart could only maintain stability for 600 cycles [165]. The rate capability of the electrode is also affected by the anion choice as electrodes with C_3_mpyrTFSI-based ILs experience a drop in capacity at rates above 2 C, while the FSI-based IL maintains 1000 mAh g^−1^ regardless of the rate. Nguyen et al. [166] investigated Si-Cu anodes with capacities of 1500 mAh g^−1^ over 200 cycles. 

#### 5.1.5. Full Cell Studies

Despite the results described above to date no full cell utilizing a Si anode, NMC or LFP cathode and C_3_mpyrFSI electrolyte has been reported. A full cell containing NMC and graphite has been investigated [161] where electrochemical impedance analysis was utilized to determine the optimum LiFSI:C_3_mpyrFSI ratio of 40:60, which offered the lowest overall cell resistance, in addition to the highest ionic conductivity. From the impedance analysis it was determined that the maximum cycling rate achievable by the cell was 0.77 C.

### 5.2. Polymer Gel Analogue

There has been extensive research carried out on C_3_mpyr-based polymer gel electrolytes where the two common cathode materials investigated are NMC and LFP. Polymer gel electrolytes that have shown compatibility with NMC electrodes consist of either C_3_mpyrFSI or C_3_mpyrTFSI. Nair et al. [167] recorded an ionic conductivity of 10^−4^ S cm^−1^ at room temperature for the polymer gel, which is one order of magnitude lower than the liquid analogue, which they attributed to the addition of the UV crosslinkers, inactive components within the gel.

#### 5.2.1. Polymers Incorporating PVDF-HFP 

By utilizing polymer materials such as poly(vinylidene fluoride-co-hexafluoropropylene) (PVDF-HFP), polymer gels with high ionic conductivities such as 3.9 × 10^−3^ S cm^−1^ at 30 °C can be obtained as reported by Singh et al. [168]. Gels consisting of PVDF-HFP, 20 wt % LiTFSI and C_3_mpyrFSI were utilized, and higher electrode capacities were obtained at a C/10 rate, where temperatures of 25 °C were employed (180 mAh g^−1^ vs. 100 mAh g^−1^) when compared to the cross-linked polymer gel synthesized by Nair et al. [167]. These results show that the incorporation of organic electrolytes within the polymer gel electrolyte is not necessary to achieve high ionic conductivities and electrode capacities. Adjusting the polymer matrix from PVDF-HFP to polyethylene oxide (PEO) can lead to significant changes in the electrochemical properties of the gel.

#### 5.2.2. Polymers Incorporating PEO 

Gupta et al. [169] utilized a PEO matrix with 20 wt % LiFSI salt and C_3_mpyrFSI, and the highest ionic conductivity of the synthesized polymer gels was 1.14 × 10^−5^ S cm^−1^, which is lower than PVDF-HFP-based polymer gels. When tested with LFP electrodes, it was determined that the interfacial kinetics were more favorable with the polymer gel electrolyte when a graphene oxide coat is applied to LFP, thus resulting in higher electrode capacities. Guerfi et al. [170] immersed LFP and Li metal electrodes in C_3_mpyrFSI and 0.7 M LiFSI with 5 wt % polymer (ether based). Heating the cell under vacuum enabled electrolyte penetration of the LFP electrode, which allowed for the formation of a stable SEI layer when cycled with Li. This resulted in capacities that were close to the rated capacity for all rates up to 2 C within an LFP/graphite full cell.

#### 5.2.3. Polymers with Coagulation or Cross-Linking 

Numerous groups have studied C_3_mpyrTFSI-based polymer gel using a variety of methods such as cross-linking [171] and coagulation [172]. Cross-linked polymer gel electrolytes such as that synthesized by Li et al. [171] have shown promising results with high electrode capacities and coulombic efficiencies (151.9 mAh g^−1^, and a coulombic efficiency of 87.9%) obtained at room temperature. After 80 cycles, an electrode capacity of 131.9 mAh g^−1^ was retained, which corresponds to a 13% capacity drop over the course of 80 cycles. These figures are impressive as elevated temperatures (>40 °C) are typically utilized in the case of other polymer gel electrolytes in order to achieve high electrode capacities.

**Figure 5:**
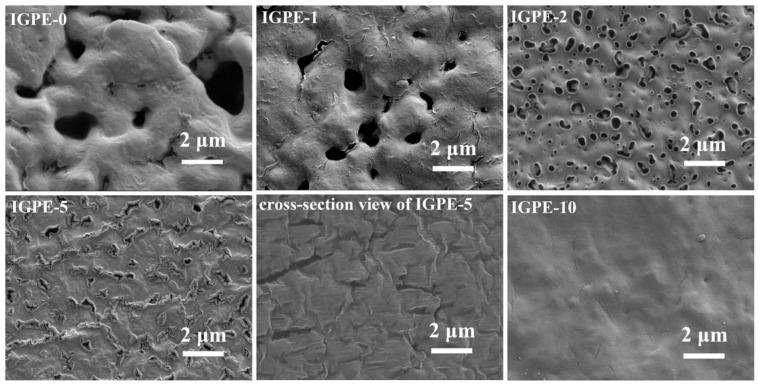
SEM images of different IGPE-X membranes, where the X refers to the length of time (in minutes) the gel spent in the coagulation bath. Reprinted with permission from Yang et al. [172].

Coagulation synthesis methods are beneficial for the formation and control of porous membranes as shown by Yang et al. [172]. By controlling the amount of time spent in the coagulation bath, the porosity of the polymer gel electrolyte can be tailored as shown in Figure 5. The porosity of the polymer gel greatly affected its ionic conductivity, with the highest values obtained for the polymer gels, which spent 5 min or less within the coagulation bath (more porous). The electrochemical tests carried out by Yang et al. [172] boasted an improved electrochemical performance when compared to the cross-linked polymer gel synthesized by Li et al. [171] as an electrode capacity of 150 mAh g^−1^ was maintained over the course of 200 cycles with no substantial capacity fade occurring over the course of 200 cycles at room temperature. Electrode capacities above 100 mAh g^−1^ were maintained even at high C-rates such as 3 C. Elevated temperatures further enhanced the electrochemical performance of the cell as electrode capacities higher than 150 mAh g^−1^, with minimal capacity fade observed over the course of 200 cycles. A similar trend was noted previously with C_3_mpyrFSI-based polymer gels, whereby the cross-linked polymer gel yielded poorer electrochemical performances when compared to the PVDF-HFP-based polymer gels.

#### 5.2.4. Polymers Incorporating Inorganic Materials

**Figure 6:**
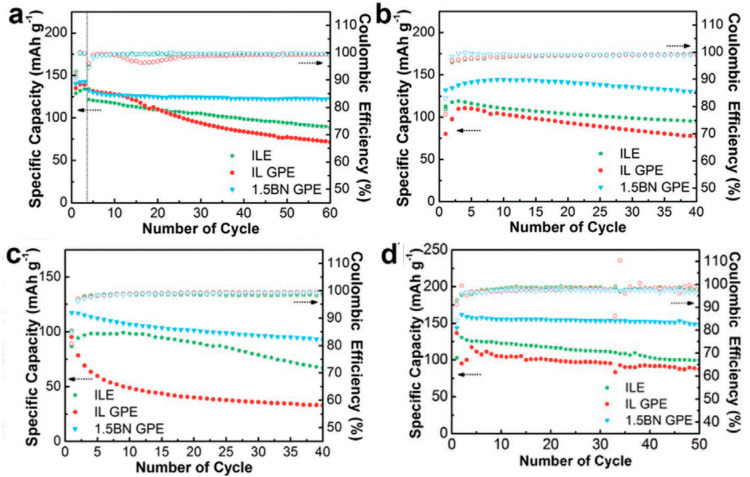
Cycling stability at 0.2 C of (**a**) LFP|Li cell, (**b**) NCM|Li cell, (**c**) LCO|Li cell and (**d**) cycling stability at 0.5 C of LFP|Li cell at 80 °C with liquid, polymer and BN-functionalized-polymer versions of C_3_mpyrTFSI. Adapted with permission from Kim et al. [173].

The utilization of inorganic materials within polymer gels has been investigated to enhance the mechanical strength, while improving ion transport properties. Kim et al. [173] investigated amine functionalized boron nitride nanosheets (AFBNNS) as an additive within a C_3_mpyrTFSI-based polymer gel. The addition of AFBNNS led to an improvement in the mechanical strength and thermal stability, in addition to yielding high Li^+^ transference numbers of 0.12–0.23, which are comparable to Yang et al. [172]. The electrochemical performance of LFP with the AFBNNS-based polymer gel electrolyte offers similar performances to Yang et al. [172], as high electrode capacities 142.4 mAh g^−1^ and 132.8 mAh g^−1^ were obtained at 0.1 C and 0.2 C, respectively. Without the presence of the AFBNNS, rapid capacity loss was observed over 60 cycles for the liquid and polymer gel electrolytes. Similarly, for NCM and LCO cathode materials, the electrochemical performance was greatly enhanced when AFBNNS-based polymer gel electrolytes were utilized. At 80 °C the LFP cell was capable of delivering a high discharge capacity of 162.1 mAh g^−1^ at the 2nd cycle with 91.3% of specific capacity retained after the 50th cycle (Figure 6).

Al_2_O_3_ has been investigated as a protective agent for Li metal and has mainly been studied from the perspective of surface coating and plating technologies. Wen et al. [174] utilized a leaf-like Al_2_O_3_ skeleton to form a quasi-solid-state electrolyte comprising of 1 M LiTFSI, and C_3_mpyrTFSI. In that instance the Al_2_O_3_ skeleton absorbed the ionic liquid, which allowed for improved ionic conductivity, in addition to promoting the migration of Li^+^ both in the bulk and at the interface. When coupled with LFP/Li full cell, the initial discharge capacity obtained was 140.7 mAh g^−1^ of which 95.6% was retained after 100 cycles at 30 °C and 0.1 C. 

C_3_mpyr-based electrolytes exhibited their versatility as they could be utilized as liquid or solid-state electrolytes (polymer gel). The cation/anion combination, C_3_mpyrFSI, has proven to be a popular choice among research groups when utilized as a liquid electrolyte as it has compatibility with high-energy density materials such as Si, which is beneficial for the miniaturization of energy storage devices. However, when utilized as a polymer gel, the more popular cation/anion combination is C_3_mpyrTFSI. The polymer gel electrolyte analysis has been limited mostly to LFP and Li metal cells, therefore further analysis is required in order to determine the compatibility of C_3_mpyrTFSI-based polymer gels with high-energy dense materials.

## 6. *N*-butyl-*N*-Methylpyrrolidinium (C_4_mpyr)-Based ILs

**Figure 7:**
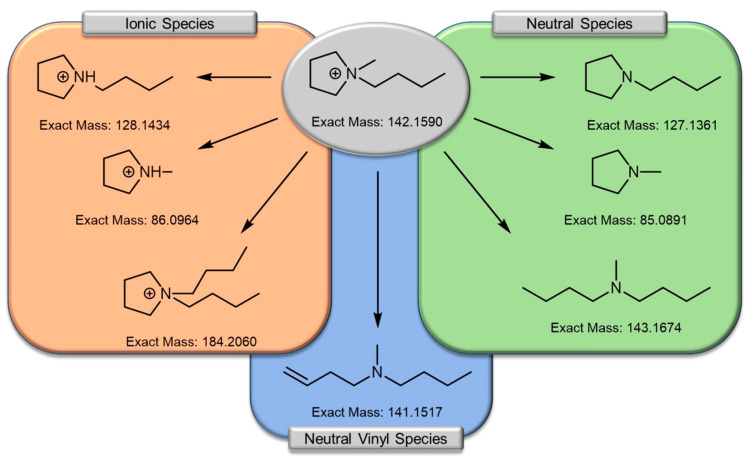
Graphical summary of the range of ionic and neutral decomposition species of C_4_mpyr-cation. The ionic species (orange box) were identified by Pyschik et al. [176,177] while Kroon et al. [178] confirmed the presence of the neutral species (green box). The neutral vinyl species was identified by Preibisch et al. [175] Reprinted with permission from Preibisch et al. [175]. Copyright 2020 American Chemical Society.

Similarly to C_3_mpyr-based ILs, there has been extensive characterization of 1-butyl-1-methylpyrrolidinium (C_4_mpyr)-based ILs [147], for example, Fernández-Miguez et al. [115] investigated high (1.5 M) and low (0.1 M) LiTFSI salt concentrations within C_4_mpyrTFSI ILs. It was determined that LiTFSI salt addition leads to higher densities than that of the pure IL, but it decreased linearly with increasing temperature. Conductivity values for pure C_4_mpyrTFSI and with various LiTFSI salt concentrations (0.1 M, 0.5 M, 1 M and 1.5 M) were reported. The ionic conductivity decreased with increasing LiTFSI salt concentration, and the authors determined the conductivity values for each concentration across a range of temperatures. Other characterization tests carried out have been mentioned in previous pyrrolidinium subsections [148,149]. Another important characterization carried out recently is the degradation of C_4_mpyrTFSI in the presence of Li metal at room temperature (Figure 7) recently studied by Preibisch et al. [175]. They identified a new degradation product *N*-butyl-*N*-methyl-*N*-but-3-eneamine, which was previously unreported and suggested that it participates in the formation of an organic layer at the surface of Li metal. This degradation product stems from the C_4_mpyr cation, rather than from the anion, which typically dominates in SEI layer formation. The presence of *N*-butyl-*N*-methyl-*N*-but-3-eneamine paves the way to the formation of potentially polymerized species that could “bind” the inorganic fraction of the decomposition layer and provide elastomeric properties to accommodate changes in the electrode volume. By harnessing the elastomeric properties of *N*-butyl-*N*-methyl-*N*-but-3-eneamine as an additive, the realization of group IV element anodes could be advanced providing the electrode volume changes can be mitigated due to the “elastic” SEI layer.

### 6.1. Liquid Electrolyte

#### 6.1.1. TFSAM-Anion Studies

The most commonly studied C_4_mpyr-based ILs are based on the TFSI^-^ and FSI^-^ anions. This is due to their superior film-forming abilities on the surface of the Li metal, which enable uniform Li deposition/dissolution with high cycling efficiencies, and no Li dendritic formation as reported by Grande et al. [179]. This study highlighted the effectiveness of Li^+^ mobility within C_4_mpyr-based ILs, which is why extensive research has been carried out using C_4_mpyr-based electrolytes with those and trifluoromethanesulfonyl-*N*-cyanoamide (TFSAM) anions. Recently, TFSAM has been utilized as an anion due to its ability to inhibit crystallization over a broader temperature range when compared to TFSI [180]. The asymmetric TFSAM anion, promotes single Li^+^ hopping due to higher structural diffusion that occurs as the LiTFSAM salt concentration increases [180]. Electrolytes containing TFSAM anions, have not been widely investigated, and present a potential area for analysis, however, C_4_mpyrTFSAM compatibility with NCM electrodes has been investigated by Hoffknecht et al. [181]. Cycling at 40 °C was carried out and high cycle efficiencies were obtained from the initial cycle, with a coulombic efficiency of 92.6%. Over 90% of the initial capacity was retained in the 30th cycle, however, the long-term stability of the cell was not described, and requires further investigation.

#### 6.1.2. Interlayers

**Figure 8:**
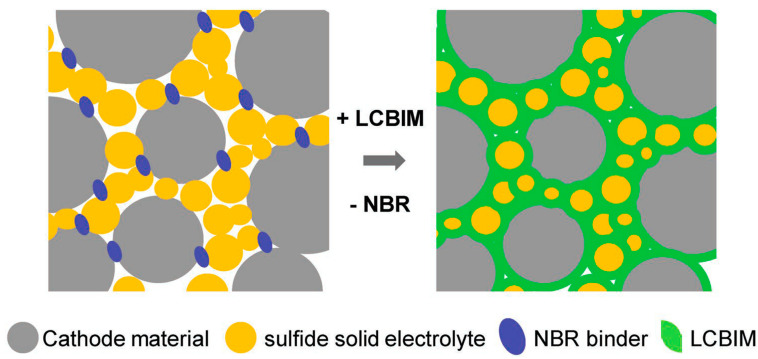
Schematic diagram representing the microstructure of the composite electrodes with polymer gel electrolyte (LCBIM), showing that LCBIM improves the interfacial contact area with conductive and adhesive properties. Carbon additives included in the composite electrode are not shown. Reprinted with permission from Cho et al. [183].

The C_4_mpyr anion combination, with TFSI and FSI anions, has again been extensively studied, not only as electrolytes, but also as electrode binders and interlayers. Liquid interlayers have been investigated at both the anode and cathode sides using C_4_mpyrFSI and LiTFSI salt. Pervez et al. [182] used it as an interlayer between the Li_6.5_La_2.5_Ba_0.5_ZrNbO_12_ (LLZO) solid-state electrolyte and the electrodes. Due to the liquid-like nature of the IL, it fills the voids between the electrode and electrolytes, and improves the interfacial properties of LLZO with both the cathode and anode. The interfacial resistance between the Li metal and LLZO electrolyte is significantly decreased upon addition of the IL interlayer. This allows for the realization of higher electrode capacities than for cells without the interlayer. Similarly, low interfacial resistance was observed for the IL interlayer in contact with LFP. Improved interfacial kinetics was observed when C_4_mpyr-based ILs were used as an electrode binder. C_4_mpyrTFSI and LiTFSI were confined within a PEO matrix to form a glue-like polymer, which then penetrated sheet-type composite NCM electrodes to act as an ion-conductive binding material, which effectively increased the effective contact area between the electrode components (Figure 8) [183]. This resulted in lower overpotentials due to the higher active area, whereby the cell achieved 92% of the capacity obtained by the NCM electrode in a conventional organic electrolyte [184].

#### 6.1.3. Cosolvents

C_4_mpyrTFSI-based electrolytes have been studied as cosolvents with conventional organic electrolytes. These electrolytes have been studied with anode materials such as graphite and Si. For instance, Balducci et al. [185] utilized C_4_mpyrTFSI as a cosolvent with LiTFSI salt, and EC/DEC/DMC (1:1:1 *w*/*w*), and observed stable cycling over the course of 50 cycles. The stability of the electrode with the electrolyte lead to minimal capacity fade over 50 cycles, and a coulombic efficiency close to 100% during cycling. These cosolvent tests showed promising results when utilized with high energy dense materials such as Si, as Balducci et al. [185] obtained stable discharge capacities of 550 mAh g^−1^ at 0.1 C, with minimal capacity fade observed across 100 cycles. The electrolyte utilized C_4_mpyrTFSI as a cosolvent with EC:DEC:DMC (1:1:1), and LiTFSI salt. 

#### 6.1.4. Sole Electrolyte

To limit the amount of organic solvents in the electrolyte, C_4_mpyr-based ionic liquid electrolytes were investigated. In liquid form, the most commonly studied C_4_mpyr-based IL is C_4_mpyrTFSI with LiTFSI salt. Utilization of an ionic liquid electrolyte can lead to lower electrode-wettability and lower Li^+^ transport number as observed by Di Lecce et al. [186]. They investigated the electrochemical performance of the olivine cathode LiFe_0.5_Mn_0.5_PO_4_ and determined that the reversible exchange of 0.6 Li could be obtained in C_4_mpyrTFSI, which corresponds to an electrode capacity of 100 mAh g^−1^. In conventional organic electrolytes, Li exchange was significantly higher at 0.7 or greater. However, this is considered a trade-off for the increased intrinsic safety of the IL electrolytes. In addition, utilization of higher operating temperatures can lead to improved performances, where the interfacial resistance decreased with increasing temperature. As the temperature increased, the electrode-wettability increased, therefore paving the way for high electrode capacities. Recently, McGrath et al. [187] reported compatibility of electrodeposited V_2_O_5_ cathode materials with C_4_mpyrTFSI-based electrolytes and Li metal anodes. Amorphous and crystalline V_2_O_5_ materials were investigated with 0.5 M LiTFSI in C_4_mpyrTFSI and 3 wt % VC in 0.5 M LiTFSI in C_4_mpyrTFSI. Superior cycling was observed for the crystalline materials, as coulombic efficiencies close to 100% were attained across all scan rates, while the amorphous material’s coulombic efficiency was scan rate dependent. Additive analysis (VC) with the crystalline material exhibited superior cycling than for its additive-free counterpart, as greater electrode capacities were attained even at 10 C (VC-containing: 75 mAh g^−1^ versus VC-free: 30 mAh g^−1^). In addition, up to 400 cycles was exhibited by the V_2_O_5_/Li cell with no evidence of electrode deterioration even at prolonged cycling at high C-rates such as 5 C.

**Figure 9:**
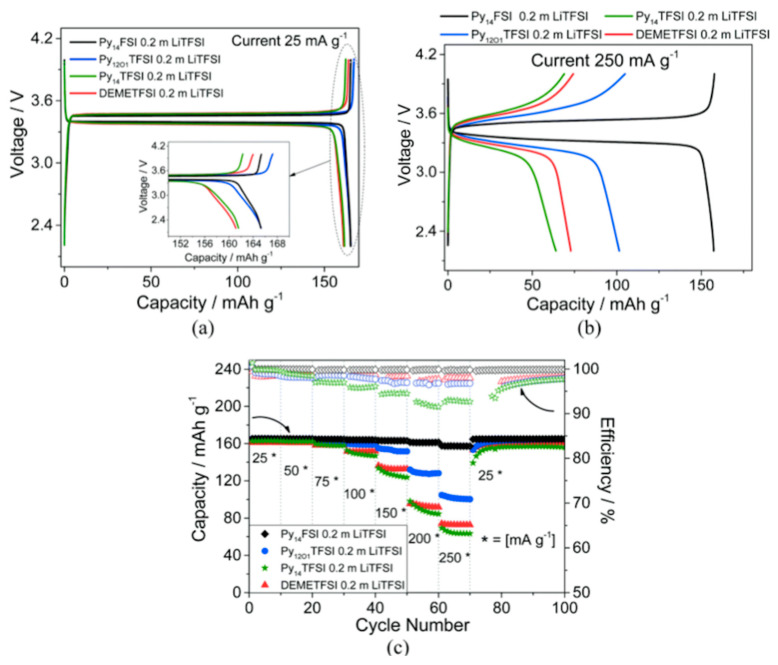
Li|IL|LFP cells galvanostatically cycled at (**a**) 25 mA g^−1^ (0.12 mA cm^−2^), with inset reporting the magnification of the final part of the curves, and (**b**) 250 mA g^−1^ (1.2 mA cm^−2^). (**c**) Cycling trend and columbic efficiency of the Li/IL/LFP cells at increasing currents. Voltage cut-offs were 2.2 and 4 V. Pyr_14_TFSI–LiTFSI (green), Pyr_14_FSI–LiTFSI (black), Pyr_1(2o1)_TFSI–LiTFSI (blue) and DEME-TFSI–LiTFSI (red). All measurements were performed at 40 °C. Reprinted with permission from Elia et al. [39].

A comparison of the electrochemical performances of C_4_mpyrTFSI- and C_4_mpyrFSI-based electrolytes has been carried out on both anode and cathode materials. Elia et al. [39] reported that C_4_mpyrFSI showed the greatest promise with LFP electrodes as it yielded the highest electrode capacity at 25 mA g^−1^ and 250 mA g^−1^ (Figure 9). The electrode capacity obtained at 250 mA g^−1^ in C_4_mpyrFSI was similar to that obtained at 25 mA g^−1^. The TFSI-based electrolyte, by comparison, gave poorer rate performance and capacities below 80 mAh g^−1^ were obtained at a current density of at 250 mA g^−1^.

A similar trend was observed for anode materials such as Ge as the lower viscosity, higher ionic conductivity C_4_mpyrFSI electrolyte enabled higher electrode discharge capacities to be obtained, 445 mAh g^−1^ vs. 260 mAh g^−1^ for C_4_mpyrTFSI. Similar trends were observed with Si electrodes [188]. The difference in the electrochemical performance was attributed to the SEI layer formation on the electrode as the anions underwent different decomposition mechanisms upon electrochemical reduction at different rates. FSI anions rapidly released F-, forming LiF in the SEI, therefore suggesting the formation of an SEI layer comprised of small inorganic compounds. TFSI anions degrade to form different SEI layer products such as -SO_2_CF_3_ at a much slower rates than FSI counterparts [189]. The composition of the SEI layer can affect the chemical structure and morphology of the SEI layer and critically influence the safety, cycle life, power capability, side reactions and shelf life of Li-ion batteries.

### 6.2. Polymer Gel Analogue

#### 6.2.1. C_4_mpyrFSI-Based Polymers

Despite the higher electrochemical performances in liquid form, the use of C_4_mpyrFSI electrolytes with polymer matrices is not commonly studied. Kerner et al. [190] synthesized PIL-based electrolytes, where one set contained C_4_mpyrTFSI, and another set contained C_4_mpyrFSI. Both electrolytes tested with LFP yielded electrode discharge capacities above 140 mAh g^−1^ at 80 °C when 0.5 C rate was utilized. The C_4_mpyrFSI containing electrolyte yielded higher electrode capacities close to theoretical (160 mAh g^−1^) for C-rates up to 0.5 C. A similar trend to the liquid electrolytes was observed as the FSI-containing electrode yielded higher electrode capacities, however, high temperatures (80 °C) were required for these high electrode capacities. By changing the PIL, Appetecchi et al. [191] synthesized films, which yielded LFP electrode capacities of 148 mAh g^−1^ (87% of the nominal capacity) at 40 °C and C/20.

#### 6.2.2. C_4_mpyrTFSI-Based Polymers

Simple polymer matrices such as PVDF-HFP, poly(ethylene carbonate) (PEC) and CMC have been investigated in addition to polymer gels, which consist of PILs. Kimura et al. [192] synthesized a polymer consisting of poly(ethylene carbonate) (PEC), LiTFSI, C_4_mpyrTFSI and non-woven SiO_2_ fiber. It exhibited low ionic conductivity at room temperature (10^−7^ S cm^−1^) and is only suited for high temperature applications. At temperatures of 75 °C, stable cycling was obtained with low electrode polarization at a C/15 rate.

Kim et al. [193] synthesized an electrospun PVDF-HFP polymer membrane, which was then soaked in 1 M LiTFSI and C_4_mpyrTFSI. An uptake of 300% was observed, which is significantly higher than that reported for Celgard separators [194]. The soaked membrane exhibited ionic conductivity of 10^−3^ mS cm^−1^ at room temperature, with a Li transport number of 0.04. Good reversibility and cyclability was observed at room temperature with LFP electrodes. Capacity retention of 92% and 69% was reported for the 0.1 C and 1 C rates after 55 cycles. The increase in electrode polarization at 1 C suggests that this membrane was better suited to C-rates below 1 C to ensure stable, long-term cycling with minimal capacity loss.

**Figure 10:**
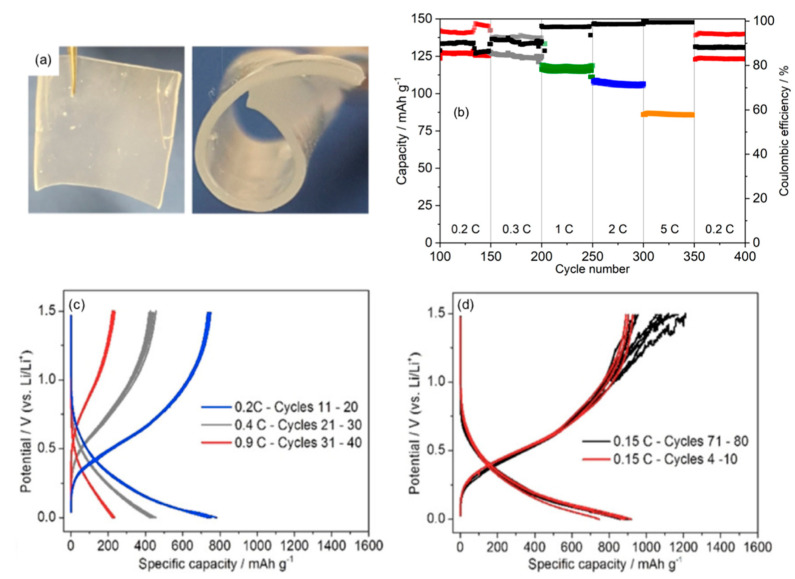
(**a**) Optical images of the 400 μm thick polymer gel synthesized, (**b**) long-term galvanostatic cycling data for V_2_O_5_/Li cells at indicated C-rates and (**c**,**d**) Ge galvanostatic profiles obtained at indicated C-rates. (**a**,**c**,**d**) Reprinted with permission from McGrath et al. [195].

An alternative to soaking a polymer membrane in the liquid IL is to encapsulate the ionic liquid in the PVDF-HFP matrix as reported recently by McGrath et al. [195]. The ionic liquid was encapsulated in the membrane and it resulted in free-standing flexible films, which exhibited ionic conductivities in the range of 7 × 10^−5^ to 1.9 × 10^−3^ S cm^−1^ at room temperature (Figure 10). Analysis with V_2_O_5_ electrodes coupled with Li metal anodes yielded promising results as 400 cycles was exhibited (Figure 10b) without capacity fade or short circuits developing, in addition, minimal capacity fade was observed when compared to its liquid counterpart (0.4 vs. 2.9% over 50 cycles at 5 C) [187]. After 400 cycles, the final 0.2 C rate capacity was recovered in the PG-60 test to 125 mAh g^−1^ after cycling at 5 C, matching the initial 0.2 C data, thus showing that that fast cycling did not lead to electrode deterioration. Its use with anode materials such as Ge has also been investigated [195]. Galvanostatic cycling analysis with Ge materials (Figure 10c,d) achieved average electrode capacities of 800 mAh g^−1^ at 0.2 C, with coulombic efficiencies greater than 99%. Capacity recovery was also observed as the 71st to 80th capacity matched that of the initial capacity at 0.15 C (900 mAh g^−1^) following cycles at higher rates. Up to 90 cycles was achieved with no capacity fade, which indicates a buffering effect of the polymer gel. 

Other anode materials have been investigated using alternative polymer matrices such as the work of Balducci et al. [185] where the conventional PVDF binder was replaced with CMC. The LTO exhibited low electrode polarization during galvanostatic cycling. The electrode capacities obtained were comparable to those exhibited by LTO with PVDF binders in conventional organic electrolytes.

### 6.3. Full Cell Studies

**Figure 11:**
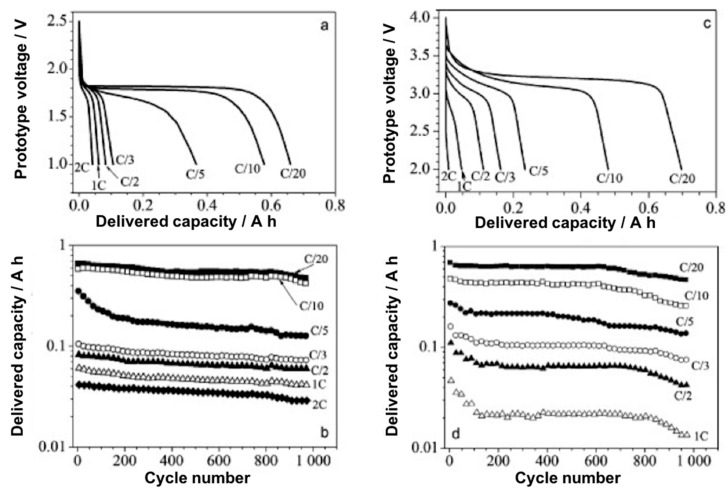
(**a**,**c**) First discharge voltage profiles and (**b**,**d**) capacity vs. cycle number at indicated discharge current rates of (**a**,**b**) LFP|0.9:0.1 C_4_mpyrFSI:LiTFSI|LTO at 20 °C (Li-ion battery) and (**c**,**d**) LFP|cl-PEO-C_4_mpyrTFSI-LiTFSI|Li at 40 °C (Li metal polymer battery). Reprinted with permission from Balducci et al. [185].

Unlike the previous pyrrolidinium cations, both liquid and polymer gel forms of C_4_mpyr-based electrolytes were utilized in battery prototype tests, where promising results were obtained. Various cathode and anode materials have compatibility with C_4_mpyr-based ILs, and therefore comprehensive full cell tests were carried out with a variety of electrode combinations. Balducci et al. [185] investigated Li metal batteries with a polymer gel electrolyte, in addition to Li-ion batteries utilizing LTP and LTO electrodes. Prototype cells were created by stacking 12–14 single bipolar cells to achieve a theoretical cell capacity of 0.7–0.8 Ah. The electrolyte utilized with the Li-ion battery was a liquid: C_4_mpyrFSI with LiTFSI salt, while the Li metal battery contained a cross-linked PEO with C_4_mpyrTFSI with LiTFSI salt. In both instances 1000 cycles were obtained for each C-rate, with minimal capacity fade observed (Figure 11). The cell capacity for both the Li metal and Li-ion batteries at C/20 was close to theoretical (0.7–0.8 Ah). The Li-ion battery shows the best electrochemical performance of the two prototype batteries, however, for low to moderate C-rates, the Li metal performance was comparable to the Li-ion battery. These results show that the presence of ILs, both solid and liquid, were suitable for use in Li-based batteries. Excellent cycle life was achieved across low to high C-rates. Increased safety was exhibited during nail puncture tests as a voltage drop was observed, however a full short circuit (voltage at 0 V) did not occur. Comparing this to a cell containing organic electrolyte, a full short circuit was achieved under the same conditions. Therefore, not only did the IL-based batteries have desirable electrochemical performances, but enhanced safety was also achieved, thus allowing for the realization of high-performance Li-based batteries. 

Similarly, Kim et al. [196] utilized Li/LFP and LTO/LFP cells with PEO-LiTFSI-C_4_mpyrFSI, and LiTFSI-C_4_mpyrFSI electrolyte, respectively. Their results reflected those obtained by Balducci et al. [185]. Full cells incorporating group IV elements with promising long-term results have been reported in the literature. Elia et al. [39] reported more than 2000 cycles with coulombic efficiency close to 100% for a Sn-C/LFP cell. From the study, it is evident that the C_4_mpyrFSI-based IL allows for good high rate capabilities, as the cell capacity was 80 mAh g^−1^ at a current density of 500 mA g^−1^. This was maintained over 2000 cycles, with a capacity retention of 98% after cycling. The exceptional cycling is due in part to the formation of an SEI layer after 10 cycles, in addition to low interfacial resistances as verified by EIS. An analysis of the SEI layer components identified SO_2_ and LiO at the anode/electrolyte interface, while other components were identified as adherent compounds such as LiF, LiO, LiOH and Li_2_SO_4_. This SEI layer differs to those typically seen in TFSI-based electrolytes as Li_2_CO_3_, Li_2_O, Li_2_S, LiF and LiOH/Li_2_O_2_ are typically observed [197]. This difference in SEI layer components allows for an improved electrochemical performance, and thus allows long-term cycling with minimal capacity fade, or cell failure.

### 6.4. Electrodeposition Medium

**Figure 12:**
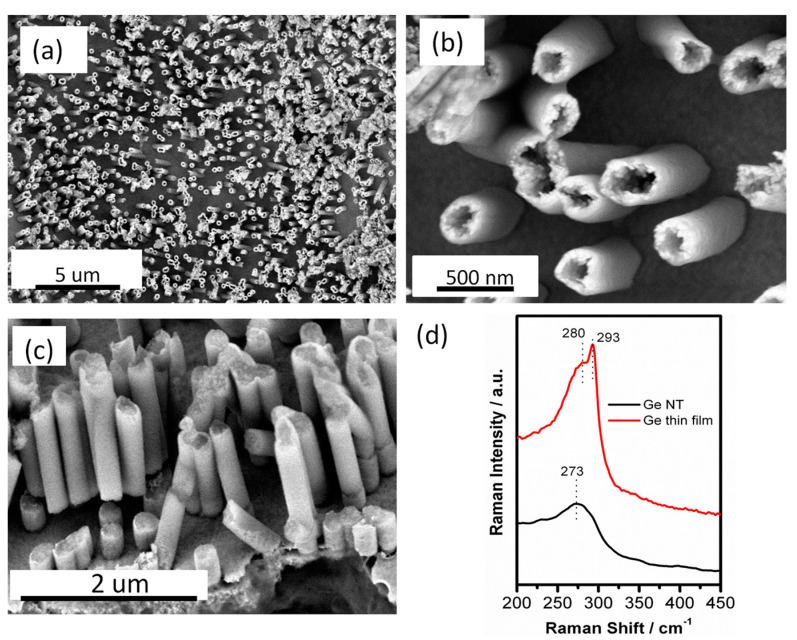
(**a**): Low magnification image of free-standing Ge nanotubes after removal of the template. (**b**) Higher magnification image of the Ge nanotubes. (**c**) Cross sectional view of the Ge nanotubes. (**d**) Raman spectra of electrodeposited Ge thin films and nanotubes obtained by exposing the sample to 5 mW, 532 nm laser for 10 s. Reprinted with permission from Lahiri et al. [201].

The above results highlight that Li-ion batteries can be realized with either liquid or polymer gel electrolytes on the cm^2^ scale. However, in order to realize battery miniaturization (mm^2^ scale) nanostructured materials are required in order to obtain high power over a small footprint, which will enable miniaturized IoT sensors. C_4_mpyr-based ILs were used to realize nanostructured materials and were utilized as the electrodeposition media for group IV elements: Sn, Ge and Si. Sano et al. [198] electrodeposited Sn onto glassy carbon electrodes using C_4_mpyrTFSI with 0.5 M C_4_mpyr Cl and 10 mM SnCl_2_. Potentiostatic cathodic reduction at −2.0 V resulted in tubular and granular Sn deposits. Similarly, electrodeposition of Ge has been achieved from a variety of C_4_mpyr-based ILs [185]. Wu et al. [199] deposited Ge from 1-butyl-1-methylpyrrolidinium dicyanamide (C_4_mpyrDCA) using [GeCl4(BuIm)_2_] (BuIm = *N*-butylimidazole) as a Ge source to create thin films. Ge deposition is also feasible at high temperatures (180 °C) to produce films [200]. Lahiri et al. [201] electrodeposited free-standing Ge nanotubes and core–shell structures through electrodeposition from C_4_mpyr-based ILs as shown in Figure 12.

Nanowire synthesis (NW) of both Ge and Si has been reported using GeCl_4_ and SiCl_4_ as the Ge and Si sources, respectively. Both Al-Salman et al. [202] and Martineau et al. [203] deposited amorphous Si-NW and Ge-NW with homogenous compositions. The capability to electrodeposit the anode material directly from ILs allows for greater electrode wettability, and therefore, there is an opportunity to explore further the range of high energy density materials that can be deposited from C_4_mpyr-based ILs. Link et al. [204] recently investigated the initial stages of Si electrodeposition from C_4_mpyrTFSI electrolytes. Their analysis suggests an instantaneous behavior of the nucleation process, showing a tendency for progressive character with decreasing the applied overpotential.

To summarize, C_4_mpyr-based ILs are versatile electrolytes in which both their liquid and polymer gel options yield promising results when coupled with a variety of cathode (LCO, NCM and LTP) and anode materials (graphite, LTO, Ge, Si and Li metal) in both half-and full-cell tests. Various full cell investigations have shown the applicability of C_4_mpyr-based ILs in both liquid and polymer form, which show that IL-based full cells are potential alternatives to conventional solid-state electrolytes. In addition, their ability to act as electrodeposition media can pave the way for nanostructured materials with enhanced electrode wettability.

## 7. Ether Functionalized Pyrrolidinium-Based ILs

### 7.1. Liquid Electrolyte

**Figure 13:**
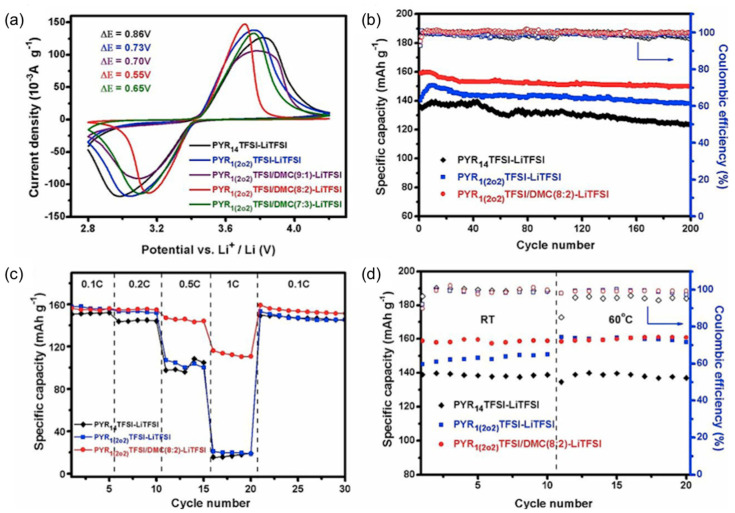
(**a**–**c**) Performance of various electrolytes at room temperature with LFP/Li: (**a**) CV curves, (**b**) cycling performance at 0.2 C, (**c**) rate performance at indicated C-rates and (**d**) cycling performance of RT and high temperature (60 °C). Reprinted with permission from Zhang et al. [209].

As with other cation types such as ammonium [37,39,40,41,42,43,44,45,46,205,206,207], research into ether functionalized ionic liquids are being investigated for pyrrolidinium-based ILs. Various ether functionalized pyrrolidinium ILs have been synthesized and characterized [148,149,208]. LFP cathodes are commonly analyzed with ether functionalized ILs as described in the previous sections and it is the cathode material of choice for the electrochemical characterization of ether functionalized pyrrolidinium-based ILs. Zhang et al. [209] designed an ether functionalized IL, 1-(2-ethoxyethyl)-1-methylpyrrolidinium bis(trifluoromethylsulfonyl) imide (PYR_1(2o2)_TFSI) in order to investigate lower viscosity, facilitate the formation of passivation layers and to enhance electrochemical stability. This IL was analyzed with LiTFSI salt, and as a cosolvent with DMC and LiTFSI salt. It was determined that the viscosity of the IL with ether functionalization was lower than for C_4_mpyrTFSI with LiTFSI. Comparing the performance with a DMC electrolyte, the ether functionalized IL electrolyte matched the electrode capacity obtained by the DMC-containing electrolyte at 60 °C, which indicates that no organic components are required for high temperature applications. Superior cycling stability and rate performance of ether functionalized ILs was exhibited when compared to the non-functionalized counterparts as capacities of 100 mAh g^−1^ and 20 mAh g^−1^, respectively, were obtained at 1 C (Figure 13).

Dual ether functionalized pyrrolidinium-based ILs have been reported by Fang et al. [210]. The electrode capacities for those functionalized pyrrolidinium-based ILs are lower than the single functionalized ILs (130–140 mAh g^−1^ vs. 150 mAh g^−1^), which shows that over-functionalization of the cations does not yield significant improvements in their electrochemical capabilities. Their physicochemical properties are significantly impacted as they offer lower viscosities, particularly P(2o1)(2o2)-TFSI, than their single- and non-functionalized counterparts (53 cP [210] versus > 100 cP at 25 °C [211]). These viscosities would allow for higher Li mobility, however, fine-tuning of the chemical structure could further improve their electrochemical abilities. These novel electrolytes show that adjustments to the chemical structure can greatly impact on its properties. This allows for further advancements in electrolyte materials and could potentially pave the way towards high powered batteries. Perhaps, IL electrolytes could achieve viscosities, and electrochemical performances similar to conventional organic electrolytes if the correct functionalized groups can be identified.

### 7.2. Polymer Gel Analogue

Polymer gel analogues have been synthesized by Ferrari et al. [212] whereby PVDF-HFP/silica composite membranes absorbed a solution of LiTFSI with *N*-ethyl(methylether)-*N*-methylpyrrolidinium trifluoromethanesulfonimide (PYRA_1(2o1)_TFSI). The composite gel electrolyte yielded promising capacity retention and reversibility with LFP cathode materials with an average coulombic efficiency of 99%. The specific capacity obtained for the gel composite was higher than for the liquid analogue over the long-term, yielding a capacity of 124 mAh g^−1^ after 174 cycles, while the liquid analogue only 89 mAh g^−1^ was achieved after 14 cycles. The compatibility of the PYRA_1(2o1)_-based gel composite electrolyte with LFP, and lower initial capacities can result in better long-term cycling as the gel composite initially had a lower capacity than its liquid counterpart (139 mAh g^−1^ vs. 156 mAh g^−1^). Further work to identify suitable ether functionalized pyrrolidinium-based ILs is required to fully utilize their potential.

## 8. Other Pyrrolidinium-Based ILs

Some pyrrolidinium cation-based ILs that have been investigated by research groups other than the cations mentioned in the previous sections include *N*-*n*-butyl-*N*-ethylpyrrolidinium (BEPyTFSI) investigated in the early 2000s in both its liquid and polymer gel forms [213,214,215], however, more recently focus has been placed on dicationic ionic liquids, which consist of an anion and a doubly charged cation as head groups linked by a variable length of alkyl or oligo ethylene glycol (OEG) chain as a rigid or flexible spacer. Mei et al. [216] synthesized a variety of dicationic-based ionic liquids and characterized their physiochemical properties. No analysis was carried out to determine their suitability for battery applications, however, they exhibited high ionic conductivities of the order 10^−3^ S cm^−1^, comparable to the single cation-based ILs.

Symmetrical cations are also being investigated such as diethylpyrrolidinium (C_2_epyr), the structure for which was shown earlier. Al-Masri et al. [217] synthesized a quasi-solid-state electrolyte containing 90 mol% LiFSI salt and C_2_epyrFSI, which exhibited an ionic conductivity of 2 × 10^−4^ S cm^−1^ at 30 °C. This value is an improvement on those outlined in Section 4, with typical ionic conductivities of 10^−6^ S cm^−1^ obtained for C_2_mpyr-based electrolytes. In addition, high Li^+^ transference numbers, and stable lithium plating and stripping behavior was reported, supported by a reduction in dendrite formation on the lithium surface by comparison with a liquid electrolyte. These results pave the way for the development of high salt content quasi-solid-state electrolytes utilizing symmetrical pyrrolidinium cations. 

## 9. Conclusions

A comprehensive review on the progress of pyrrolidinium ILs was outlined. From the discussion, there are significant areas for improvement within the pyrrolidinium family. For instance, the lack of research into compatible cathode and anode materials with C_2_mpyr-based electrolytes could be an area of interest as only NCM, LFP, LTO and Li metal have been investigated to date. Considering their ability to encapsulate the electrode particles, effectively decreasing the internal resistance of the battery, and their ability to hamper Li dendritic growth [32] C_2_mpyr-based electrolytes are excellent candidates for future Li-battery technology. Similarly, further research can be carried out for C_3_mpyr-based polymer electrolytes as analysis has been limited mostly to LFP and Li metal cells. Liquid versions of C_3_mpyr-based ILs have shown compatibility with Si electrode materials, and creation of a polymer gel analogue with similar compatibility could pave the way for high-energy dense Li-batteries.

Recent advancements into ether_-_functionalization of the cations demonstrate further applicability as novel electrolytes can be identified. For instance, the duel ether-functionalized electrolytes yielded capacities of 130–140 mAh g^−1^ [210], which is similar to their single-functionalized counterparts, however, their viscosity is significantly lower at 53 cP [210] versus >100 cP at 25 °C [211]. They have the potential to achieve viscosities similar to organic-based electrolytes, which would make them competitive as a safe alternative, provided they match the electrochemical performance of the organic electrolytes. Another area that will allow for further development is in the synthesis of new compatible anion materials such as TFSAM. Utilization of different anions will allow for more electrolyte tunability. For example, the asymmetric TFSAM anion promotes single Li^+^ hopping due to higher structural diffusion and is capable of inhibiting crystallization over a broader temperature range when compared to TFSI [180]. In addition, alternative anions can be utilized to synthesize ILs suitable for electrodeposition, such as C_4_mpyrDCA, which has been utilized to deposit Ge thin films [199]. 

Currently, microbattery research with ILs has been limited. This is attributed in part to the lack of understanding of the roles the cations, anions and lithium salt play in the electrochemical and physicochemical properties of ionic liquid electrolytes. This is being addressed by various research groups, where new breakdown products such as *N*-butyl-*N*-methyl-*N*-but-3-eneamine are being discovered [175]. *N*-methyl-*N*-but-3-eneamine has the potential to provide elastomeric properties to accommodate changes in the electrode volume, which could facilitate the use of group IV materials in commercial microbatteries, thus enabling their use within healthcare applications such as rechargeable implantable devices where high power is required for data transmission. Studies with nanostructured V_2_O_5_ (nanoribbon, nanowire and microflake) have indicated promising electrochemical performances with C_3_mpyrTFSI electrolytes [160]. Coupling this with their ability to deposit electrode materials in-situ can enable nanostructured active materials for microbatteries with high electrode wettability, to deliver high power in a small footprint. Promising long-term cycling of Li metal with pyrrolidinium-based IL where no short circuiting is observed after 400 cycles in both liquid and polymer gel forms [187] further indicates the applicability of ILs for microbatteries. 

Ionic liquids are promising alternative electrolyte materials to both solid-state and organic electrolytes, due to their superior electrochemical and physicochemical properties, in addition to their compatibility with a wide array of cathode and anode materials. They are extremely versatile materials as they have been utilized as interlayers, additives, cosolvents, a component in mixed IL electrolytes, the sole electrolyte and as polymer gels. Their versatility is clearly highlighted in the preceding sections, with each cation offering properties, which may be tailored for specific applications. Further investigation into their properties and capabilities will allow for improvements in the miniaturization of energy storage devices for portable or miniaturized electronic devices such as sensors for the IoT.

## Figures and Tables

**Table 1:** Structures of pyrrolidinium cations and commonly used anions discussed in this review.

Cation Structure	Name	Abbreviations
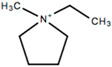	*N*-ethyl-*N*-methylpyrrolidinium	C_2_mpyr, C_2_PYrr, P_12_
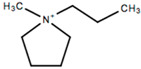	*N*-propyl-*N*-methylpyrrolidinium	C_3_mpyr, C_3_PYrr, P_13_, PYR_13_, PMP, PMPyr
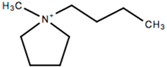	*N*-butyl-*N*-methylpyrrolidinium	C_4_mpyr, C_4_PYrr, P_14_, PYR_14_, BMP, BMPyr
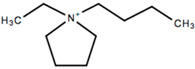	*N*-n-butyl-*N*-ethylpyrrolidinium	BEPy, P_24_
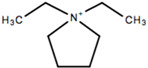	Diethylpyrrolidinium	C_2_epyr
**Functionalized pyrrolidinium cations**		
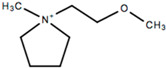	*N*-ethyl(methylether)-*N*-methylpyrrolidinium	PYRA_1(2o1)_
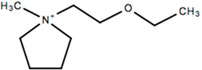	1-(2-ethoxyethyl)-1-methylpyrrolidinium	PYR_1(2o2)_
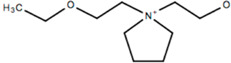	*N*-(methoxyethyl)-*N*-(ethoxyethyl)pyrrolidinium	P(2o1)(2o2)
**Anion Structure**	**Name**	**Abbreviations**
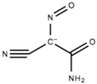	Carbamoylcyano(nitroso)methanide	ccnm
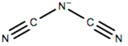	Dicyanamide	DCA
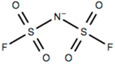	Bis(fluorosulfonyl)imide	FSI, FSA
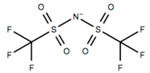	Bis(trifluoromethylsulfonyl)imide/Bis(trifluoromethane)sulfonimide	TFSI, TFSA, NTf_2_
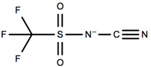	Trifluoromethylsulfonyl-*N*-cyanoamide	TFSAM
